# Mildly Low Serum Sodium Levels in Chronic Liver Disease: At Risk for Sarcopenia and Portal Hypertension

**DOI:** 10.7759/cureus.44419

**Published:** 2023-08-30

**Authors:** Atsushi Nakamura, Tsubasa Yoshimura, Takeshi Ichikawa

**Affiliations:** 1 Hepatology, Nippon Koukan Hospital, Kawasaki, JPN; 2 Gastroenterology, Nippon Koukan Hospital, Kawasaki, JPN

**Keywords:** sarcopenia, liver stiffness, portal hypertension, magnetic resonance elastography, serum sodium level

## Abstract

Objective: Hyponatremia and sarcopenia in advanced chronic liver disease (ACLD) are both associated with portal hypertension (PHT) and worse prognosis. This study investigated their interrelationship.

Methods: This retrospective study analyzed 751 patients with CLD who underwent magnetic resonance elastography (MRE) at Nippon Kokan Hospital (Kawasaki, Japan). Patients were classified and studied in five groups based on serum sodium (Na) levels: <135, 135-136, 137-138, 139-140, and >140 mEq/L. PHT was assessed by thrombocytopenia, varices, and ascites, and magnetic resonance imaging (MRI) data were used to diagnose sarcopenia.

Results: The proportions of the five groups were 3/4/13/32/48 (%), and the mean liver stiffness (LS) was 6.6/5.7/4.2/3.2/3.2 (kPa), with significant progressive increases at Na < 139 (*p*< 0.01). The incidence of all PHT events and sarcopenia also increased at <139 (each *p* < 0.01). By contrast, the LS thresholds for predicting thrombocytopenia, varices, and ascites increased from 3.5 to 4.7 and 5.1, respectively, and were the same at 3.4 for low Na (<139) and sarcopenia (all *p* < 0.01). Multivariate analysis of factors associated with low Na identified LS and sarcopenia as independent factors (*p* < 0.05 both). In the Cox proportional hazards model, low Na was a significant prognostic factor in ACLD (hazard ratio (HR) 5.33, *p* < 0.01); however, the albumin-bilirubin (ALBI) score (HR 2.49) and sarcopenia (HR 4.03) were extracted in the multivariate analysis (*p* < 0.05 both).

Conclusions: Studies using MRE imaging showed that low Na levels in CLD are associated with worse prognosis, not only due to elevated LS (i.e., PHT) but also the strong association with sarcopenia.

## Introduction

Advanced chronic liver disease (ACLD) presents with hyponatremia caused by impaired electrolyte and fluid homeostasis. Sodium (Na) is essential for the regulation of blood volume, blood pressure, osmotic balance, and blood pH, and hyponatremia is defined as a serum Na level <135 mEq/L. However, in cirrhosis, the severity of hyponatremia is associated with the severity of liver disease and poor prognosis [1,2]; thus, the new Model for End-Stage Liver Disease (MELD) score incorporates a wide range of serum Na levels (125-140 mEq/L), including even normal values [3]. Hyponatremia in ACLD has a complex and multifactorial pathogenesis. First, portal hypertension (PHT) caused by advanced liver fibrosis triggers splanchnic vasodilation. Then, a decrease in effective arterial blood volume not only induces antidiuretic hormone secretion but also activates the renin-angiotensin-aldosterone system and the sympathetic nervous system, causing water and Na retention in the kidney, ultimately resulting in dilutional hyponatremia [4]. In cirrhosis with ascites, nearly all guidelines recommend moderate salt restriction (5-6 g/day). However, in clinical practice, the pros and cons of salt-restricted diets are controversial mainly because a salt-restricted diet may cause anorexia and exacerbate malnutrition in cirrhosis. Protein and energy malnutrition is present in 50-90% of patients with cirrhosis and is an important factor associated with sarcopenia [5].

In the general population, drugs, such as diuretics, antidiuretic hormone deficiency, cancer, and malnutrition, cause chronic hyponatremia. However, because most cases of chronic hyponatremia are asymptomatic, it has received little attention [6]. Recently, it has been shown that mild hyponatremia in the elderly is associated with falls, fractures, and poor prognosis, and the relationship between chronic hyponatremia and sarcopenia and life expectancy has attracted attention [7,8,9]. Other studies in rats have shown an association between chronic hyponatremia and hypogonadism and muscle atrophy [10], and human clinical studies have reported an independent association between mild hyponatremia and sarcopenia [11]. Meanwhile, in cirrhosis, sarcopenia, a phenotype of malnutrition, is considered a prognostic factor independent of the hepatic reserve function. The pathogenesis of sarcopenia in liver disease is also thought to be a complex interplay of various factors. Among them, nutritional disturbance caused by PHT is one of the most important factors [12,13]. Therefore, we were interested in the relationship between low serum Na levels and sarcopenia in CLD, which has not been investigated to date.

At present, magnetic resonance elastography (MRE) is considered the optimal noninvasive test (NIT) for the diagnosis and staging of liver fibrosis, as evidenced by multiple etiologic studies [14,15]. Furthermore, liver stiffness (LS) on MRE measurement has been reported to be useful in predicting the risk of PHT and prognosis in ACLD [16,17]. In addition, the measurement of muscle mass on MR images has been reported to be useful in the diagnosis of sarcopenia [18,19]. This study aimed to determine the association between low serum Na levels and sarcopenia (i.e., muscle mass loss) in CLD using MRE imaging data analysis.

## Materials and methods

Study population

In this study, 751 of 773 patients with CLD who underwent MRE at Nippon Kokan Hospital (Kawasaki, Japan) between July 1, 2017 and January 31, 2023 were included in the analysis. MRE was conducted to diagnose liver fibrosis and hepatic steatosis and the presence of liver tumors on non-contrast magnetic resonance imaging (MRI). Excluded from the analysis were 12 patients whose height or weight was not recorded, four for whom elastography was difficult because of massive ascites or excessive iron deposits, and six whose muscle area could not be measured because of metal artifacts from spinal fusion surgery. The CLD etiologies were hepatitis B, hepatitis C, non-alcoholic fatty liver disease (NAFLD), alcoholic liver disease, autoimmune liver disease, and unknown in 153, 169, 229, 109, 48, and 43 patients, respectively. All patients with hepatitis C who achieved sustained viral response with the treatment were included. This study was approved by the Ethical Review Board of Nippon Koukan Hospital (approval no. 202014). The study was conducted in accordance with the “Ethical Principles for Medical Research Involving Human Subjects” described in the 1975 Declaration of Helsinki as revised in 2000. Informed consent of the participants was obtained in an opt-out manner.

Data collection

Parameters of age, gender, height, weight, body mass index (BMI), and baseline laboratory data were collected for each patient. The mean and median intervals between clinical data and MRE dates used in the analysis were 9 ± 10 days and 7 (0-16) days, respectively. The definition of ACLD in this study was F3 and F4 patients diagnosed by MRE, and the severity of liver dysfunction was assessed by the albumin-bilirubin (ALBI) score [20] and MELD-Na score [3]. In addition, the progression of ACLD was stratified by the modified ALBI (mALBI) grade [21]. The ALBI score is an index of hepatic reserve developed to assess the prognosis of patients with HCC. The ALBI score was calculated using the formula and classified into grade 1 (ALBI score < -2.60), grade 2 (-1.39 < ALBI score < -2.60), and grade 3 (ALBI score > -1.39). Grade 2 was further divided into grade 2a (-2.60 < ALBI score < -2.27) and 2b (-2.27 < ALBI score < -1.39). Thus, mALBI was classified into four grades: G1, G2a, G2b, and G3.

$ALBI score = (log10 bilirubin[&mu;mol/L] &times; 0.66) + (albumin [g/L] &times; -0.085)$

Diagnostic Criteria for the Etiology of CLD and Diagnostic Procedures for Cirrhosis and Hepatocellular Carcinoma (HCC)

Alcoholic liver disease was diagnosed according to the diagnostic criterion of the Japanese Society for Biomedical Research on Alcohol [22], and NAFLD was diagnosed according to imaging findings (hepatic and renal contrast on abdominal ultrasonography; liver/spleen ratio, <0.9 on abdominal CT; MRI-proton density fat fraction (PDFF), >5.2% [23]) and the lack of alcohol overuse (pure ethanol equivalent <30 and <20 g/day for males and females, respectively). Cirrhosis was diagnosed based on histological or clinical findings. Clinical findings include typical ultrasound findings, low platelet counts (<100,000/μL), and complications, such as varices. HCC was diagnosed according to the guidelines [24,25] as tumors marked in the arterial phase on contrast-enhanced CT and showing washout in the portal or delayed phase. In the case of gadoxetate disodium (EOB) contrast-enhanced MRI, tumors were also defined as those that stained in the early phase and showed washout in the portal venous phase.

MRE Protocol

In our institution, MRE was performed as an add-on to conventional non-contrast MRI. All the patients fasted overnight (>12 h) before evaluation using a 1.5-T whole-body MRI system (SIGNA Voyager XT 1.5T; GE Medical Systems, Milwaukee, WI, USA). LS was measured by elastography to determine hepatic fibrosis progression [26], whereas intrahepatic fat content was measured using the iterative decomposition of water and fat with echo asymmetry and least squares estimation (IDEAL-IQ) as proton density fat fraction (PDFF) [27]. LS (kPa) and PDFF (%) were analyzed by a radiologist skilled in liver imaging. Methods and criteria for liver fibrosis classification by MRE are described below. 

Liver Fibrosis Classification Methods and Criteria

The diagnosis of F stage was made according to two criteria for each etiology, considering that liver stiffness measurement (LSM) on MRE differed according to etiology. NAFLD was diagnosed using the criteria of Hsu et al. [28]: fibrosis stages 0 through 4, with thresholds of 2.61, 2.97, 3.62, and 4.69 kPa, respectively. The other causes were diagnosed using Morisaka et al.’s criteria [29]: threshold values of 2.32, 2.61, 3.02, and 4.23 kPa.

Nutritional Assessment of Patients With CLD

In this study, malnutrition was assessed by sarcopenia and the Controlling Nutritional Status (CONUT) score [30], an objective nutritional assessment measure. Skeletal muscle mass was measured using MRI images according to previous reports [18,19]. In practice, the paraspinal muscle area was measured at the level of the superior mesenteric artery on MRI images, and a cut-off value of 12.62 cm^2^/m^2^ in males and 9.77 cm^2^/m^2^ in females was applied to diagnose sarcopenia. The CONUT score is an objective nutritional assessment scoring system based on the calculation of three items: serum albumin level, peripheral blood total lymphocyte count, and total cholesterol level.

Evaluation of PHT 

In this study, thrombocytopenia (platelet count < 15 × 10^4^/mm^3^) [31] and gastric or esophageal varices (≥F1 or with red signs) were used to evaluate PHT. Varices were diagnosed in 444 cases, in which esophagogastroduodenoscopy (EGD) was performed within one year after MRE (mean interval 70 ± 79 days, median 53 days). Hepatic decompensation was diagnosed by ascites (grade ≥ 1) [12] on MRI. In addition, patients with ascite complications received two types of treatment: lupus diuretics and anti-aldosterone diuretics.

Statistical analysis

JMP statistical software version 12.2 (SAS Institute Japan, Tokyo, Japan) was used in all statistical analyses. The chi-square test, Wilcoxon-Mann-Whitney test, and Spearman’s rank correlation coefficient were used for the inter-group analyses, and multiple comparisons of variables among multiple groups were performed using the Steel tests after significant differences were confirmed by the Kruskal-Wallis test. In this study, receiver operating characteristic (ROC) analysis was used to identify the respective cutoff values of LS and serum Na levels that predict the onset of each liver disease-related event. Multiple regression analysis and logistic regression analysis was used to examine factors associated with low serum Na levels and sarcopenia in CLD. A stepwise increase/decrease method was used to select variables. Patient prognosis was analyzed using Kaplan-Meier and Cox proportional hazards methods, and the stepwise decrease method was used for variable selection. Statistical significance was set at a p-value of <0.05.

## Results

Baseline characteristics

In this study, the 751 subjects were divided into five groups (<135, 135-136, 137-138, 139-140, and >140) according to serum Na levels (mEq/L): 19, 34, 95, 238, and 355, respectively. The comparative analysis of the groups showed significantly more males and alcoholic liver disease in the low Na group. In addition, severity of liver damage, renal damage, and ALBI score were each significantly associated with low Na levels, but not with ammonia. Low Na levels were also significantly associated with the neutrophil-lymphocyte ratio (NLR) and C-reactive protein (CRP), which are indicators of systemic inflammation.

The characteristics of all patients are shown in Table 1.

**Table 1:** Clinical characteristics at baseline Statistics are shown as mean ± SD (standard deviation) or n (%). ALBI, albumin–bilirubin grade; ALP, alkaline phosphatase; ALT, alanine aminotransferase; AST, aspartate aminotransferase; BMI, body mass index; BUN, blood urea nitrogen; CONUT, controlling nutritional status; CRP, C-reactive protein; γ-GTP, γ-glutamyl transpeptidase; HCC, hepatocellular carcinoma; NLR, neutrophil-to-lymphocyte ratio; PT, prothrombin activity; TC, total cholesterol

		Serum sodium levels (mEq/L)					
	All cases	< 135	135-136	137-138	139-140	> 140	
	(751)	(20)	(34)	(98)	(242)	(357)	p-value
Age (y)	62±15	69±11	65±13	62±17	61±15	63±14	0.150
Sex (M/F)	449/302	14/6	26/8	68/30	147/95	194/163	0.019
BMI (kg/m^2^)	24.5±4.3	24.3± 7.4	23.2± 4.0	24.3± 4.2	24.9± 4.2	24.4± 4.3	0.271
Alcoholic liver disease	104 (14)	10 (53)	14 (41)	16 (17)	18 (8)	46 (13)	< .001
Leucocytes (/mm^3^)	5782±1716	7955±4122	6419±3709	5726±1692	5791±1557	5730±1458	0.024
Hemoglobin (gl/dl)	13.8±1.9	12.3±2.5	12.9±2.5	13.4±2.4	13.8±1.7	14.1±1.8	< .001
Platelets (x10^4^/mm^3^)	20.1±6.7	14.6±7.3	17.6±7.6	18.9±7.7	20.5±6.4	20.7±6.3	< .001
Total bilirubin (mg/dL)	1.0±0.9	1.9±1.8	1.4±1.0	1.1±1.0	1.0±1.1	0.9±0.7	0.013
AST (IU/L)	41±84	70±64	89±216	42±29	39±44	42±110	< .001
ALT (IU/L)	48±146	35±23	100±378	39±32	43±62	49±162	0.401
Al-P (IU/L)	284±181	421±229	351±188	264±155	269±220	281±150	< .001
γGTP (IU/L)	95±177	167±268	184±341	148±321	73±117	90±126	0.001
Albumin (g/dl)	4.1±0.5	3.1±0.9	3.5±0.8	4.0±0.7	4.2±0.4	4.2±0.4	< .001
Total cholesterol (mg/dL)	193±42	151±59	166±51	184±43	196±40	198±39	< .001
Trigliceride (mg/dL)	148±116	112±94	150±160	159±122	143±115	148±108	0.046
BUN (mg/dL)	16±8	18±9	22±24	17±10	15±6	14±6	0.112
Creatinine (mg/dL)	0.8±0.7	0.9±0.3	1.0±0.8	1.1±1.5	0.8±0.3	0.8±0.3	0.038
Sodium (mEq/L)	140±2.9	129±6.7	136±0.5	138±0.5	140±0.5	142±1.1	< .001
Prothrombin activity (%)	97±17	76±21	84±18	92±16	97±18	99±15	< .001
Ammonia (μg/dL)	37±29	44±29	53±39	41±43	34±27	34±24	0.118
CRP (mg/dL)	0.3±1.1	2.2±4.2	0.8±1.2	0.4±0.8	0.3±1.0	0.2±0.4	< .001
NLR	2.01±1.57	6.44±6.80	2.20±1.25	2.18±1.17	1.80±0.92	1.87±1.02	< .001
HCC	60 (8)	5 (26)	4 (12)	11 (12)	17 (7)	23 (7)	0.057
Diuretics	43 (6)	7 (37)	7 (21)	11 (12)	10 (4)	8 (2)	< .001
ALBI score	-2.24±0.68	-1.81±0.82	-2.14±0.76	-2.57±0.65	-2.79±0.42	-2.80±0.41	< .001

Association of serum Na levels with LS (kPa) in CLD

**Figure 1:**
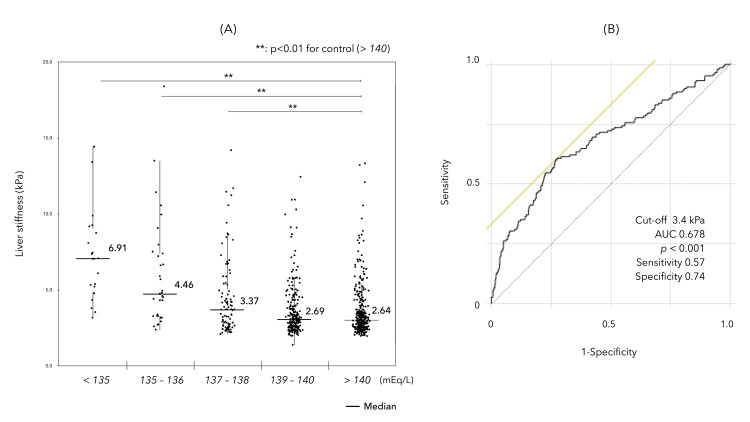
Comparison of LS values, portal hypertension-related events, and sarcopenia in the five groups (<135, 135–136, 137–138, 139–140, and ≧140 mEq/L) based on the serum sodium level (Na). No difference was found in the LS values (A) between the 139–140 and >140 mEq/L groups, and in the Na < 139 mEq/L group, increased LS was significantly associated with decreased Na levels (*p* < 0.01 by Steel tests). (B) The cut-off value for LS predicting low Na (<139) by the receiver operating characteristic (ROC) curve analysis was 3.4 kPa.

The mean LS measured by MRE in CLD was 3.54 ± 2.18 kPa, and the mean LS values in each of the five groups by serum Na levels were 6.63 ± 2.74, 5.73 ± 3.72, 4.15 ± 2.63, 3.23 ± 1.75, and 3.20 ± 1.76, with a significant progressive increase in the serum Na level < 139 mEq/L. The ROC analysis showed that the LS cut-off values that predicted low Na (< 139 mEq/L) were 3.4 kPa (Figure 1A, 1B).

Liver disease-related events and serum Na levels in CLD

**Figure 2:**
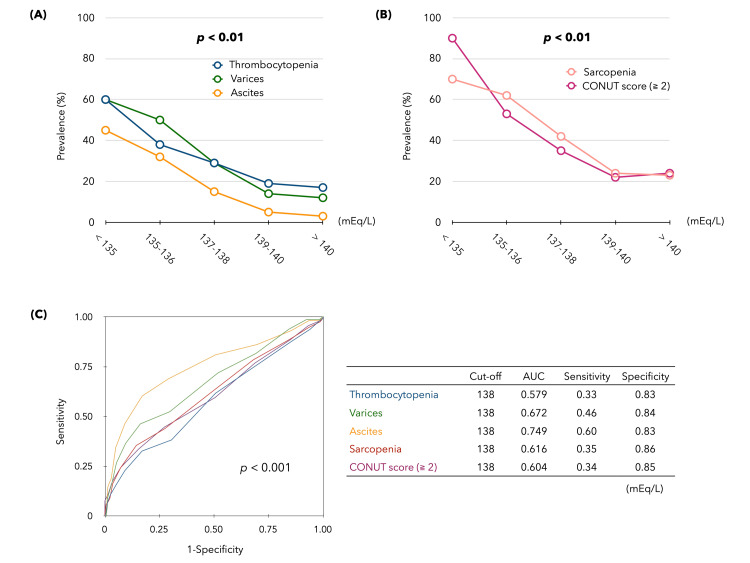
Prevalence of each liver disease-related complication by the serum Na level in CLD. (A) Prevalence of each portal hypertension (PHT)-related event in the five groups based on serum Na levels. (B)  Prevalence of malnutrition (sarcopenia and CONUT score ≥ 2) in the five groups based on serum Na levels. (C) Receiver operating characteristic (ROC) curves for each serum Na level (mEq/L) predicting the occurrence of five liver-related complications.

The prevalence of PHT-related events in CLD was 21.2% (162/751) for thrombocytopenia, 18.5% (82/444) for varices, and 7.7% (58/751) for ascites. Meanwhile, sarcopenia in CLD was found in 29% of the patients and the CONUT score ≥2 in 28% of the patients.The prevalence of the three PHT-related events was significantly increased at serum Na levels < 139 mEq/L (Figure 2A). Patients with sarcopenia and CONUT score ≥2, indicators of malnutrition, also showed a significant progressive increase in serum Na levels < 139 mEq/L (Figure 2B). In the ROC analysis (Figure 2C), the cutoff values of serum Na levels predicting each of the five events were all 138 mEq/L, with a sensitivity of 0.33-0.60 but a high specificity of 0.83-0.86 (*p *< 0.001).

Liver disease-related events and LS in CLD

**Figure 3:**
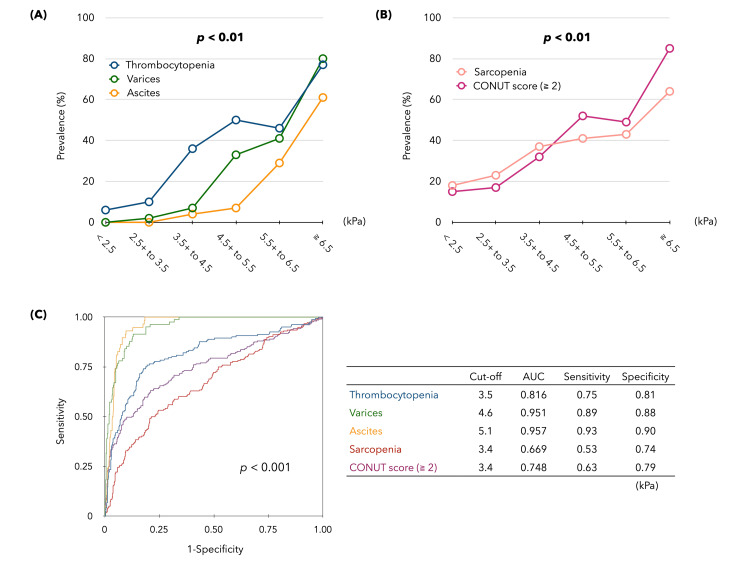
Prevalence of each liver disease-related complication by LS in CLD. (A) Prevalence of PHT-related event classified into six groups based on LS (kPa). (B) Prevalence of malnutrition (sarcopenia and CONUT score ≥2) in the six groups based on LS (kPa). (C) Receiver operating characteristic (ROC) curves of LS (kPa) that predict the occurrence of each liver-related event.

The patients were divided into six groups based on LS (from <2.5 kPa to ≥6.5 kPa in increments of 1.0 kPa), and the prevalence of each event was examined (Figures 3A, 3B). The prevalence of all liver disease-related events was significantly associated with an increase in LS, but there were differences in the LS values as the starting point for the development of each event. ROC analysis (Figure 3C) showed that the cutoff values of LS (kPa) predicting the occurrence of each PHT-related event were 3.5 kPa for thrombocytopenia, 4.7 kPa for any varices, and 5.1 kPa for ascites (≥grade 1), indicating that LS elevation was proportional to PHT severity. By contrast, in patients with sarcopenia and CONUT score ≥2, indicating nutritional impairment, the LS threshold was 3.4 kPa, the same as the LS threshold for low Na. 

Multivariate analysis of factors associated with serum Na levels in CLD

The univariate analysis showed significant associations with serum Na levels in the following parameters: sex, etiology (alcoholic liver disease), total bilirubin, aspartate aminotransferase (AST), alanine aminotransferase (ALP), γ-glutamyl transpeptidase (γ-GTP, albumin, total cholesterol, blood urea nitrogen (BUN), creatinine, prothrombin activity, NLR, CRP, LS, HCC, ascites, diuretic use, and ALBI score (*p* < 0. 05). Factors that were significant in the univariate analysis were utilized in the multivariate analysis. The multiple regression analysis then included eight factors selected by the stepwise method (Table 2). LS, NLR, BUN, and sarcopenia were identified as independent factors contributing to lower serum Na levels in CLD. 

**Table 2:** Multiple regression analysis of factors associated with serum Na levels in CLD. AST, aspartate aminotransferase; BUN, blood urea nitrogen; CONUT, controlling nutritional status; γ-GTP, γ-glutamyl transpeptidase; LS, liver stiffness; NLR, neutrophil-to-lymphocyte ratio

	*β* (95% CI)	*p*-value
LS (kPa)	-0.17 (-0.32, -0.08)	< .001
NLR	-0.16 (-0.43, -0.12)	< .001
Sarcopenia	-0.12 (-0.65, -0.09)	0.011
BUN (mg/dL)	-0.10 (-0.06, -0.00)	0.031
Alcoholic liver disease	-0.08 (-0.64, 0.02)	0.069
CONUT score	-0.09 (-0.26, 0.03)	0.114
Platelets (x10^4^/mm^3^)	-0.07 (-0.07, 0.01)	0.138
Sex (F)	0.03 (-0.16, 0.33)	0.484

Additional analysis of the relationship between sarcopenia and low Na

**Figure 4:**
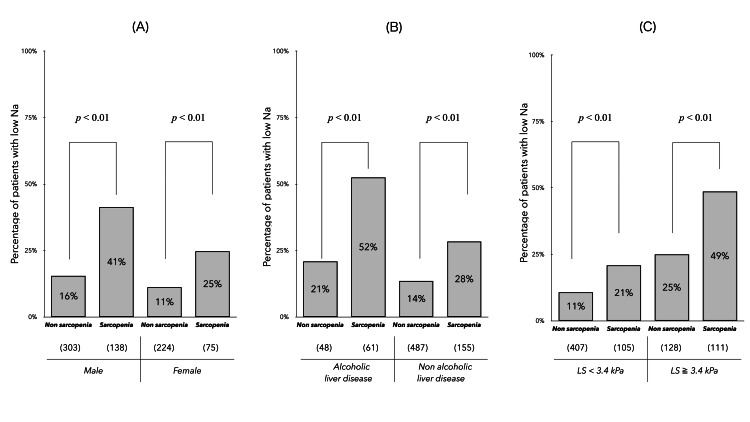
Additional analysis of the relationship between sarcopenia and low serum Na levels (<139 mEq/L) in the patients with CLD. (A) The prevalence of sarcopenia in patients with and without low Na levels was compared by sex. (B) The prevalence of sarcopenia in patients with and without low Na levels was compared by etiology (alcoholic liver disease vs. other etiologies. (C) The prevalence of sarcopenia in patients with low Na levels was compared by the liver fibrosis stage (LS: < 3.4 kPa or ≥ 3.4 kPa).

The association between sarcopenia and low Na (<1.39 mEq/L) in CLD (Figure 4) was found irrespective of the degree of liver fibrosis (LS < 3.4 kPa or ≥ 3.4 kPa). Therefore, the relationship between the two was analyzed only in patients with LS < 3.4 kPa who were unaffected by PHT. Multivariate analysis identified low Na as an independent factor associated with sarcopenia, together with age and malnutrition (BMI and CONUT score) (Table 3).

**Table 3:** Additional univariate and multivariate analyses of factors associated with sarcopenia in CLD patients with LS < 3.4 kPa only. BMI, body mass index; ALT, alanine aminotransferase; CONUT, controlling nutritional status

	Univariate analysis		Multivariate analysis	
	Odds ratio (95% CI)	p-value	Odds ratio (95% CI)	p-value
Age (y)	1.05 (1.04-1.07)	< .001	1.06 (1.03-1.08)	< .001
BMI (kg/m^2^)	0.83 (0.79-0.87)	< .001	0.81 (0.74-0.87)	< .001
Alcoholic liver disease	3.38 (1.70-6.62)	< .001		
Leucocytes (/mm^3^)	0.71 (0.65-0.78)	< .001		
Hemoglobin (gl/dl)	0.83 (0.73-0.95)	0.005	1.15 (0.98-1.37)	0.109
Platelets (x10^4^/mm^3^)	0.94 (0.90-0.98)	< .001		
ALT (IU/L)	0.99 (0.98-0.99)	0.014		
Albumin (g/dl)	0.31 (0.16-0.61)	< .001		
Sodium (<139 mEq/L)	3.28 (2.27-4.75)	< .001	2.05 (1.05-3.92)	0.035
NLR	1.37 (1.08-1.75)	0.010		
CONUT score	1.64 (1.34-2.01)	< .001	1.55 (1.23-1.99)	< .001

Analysis of prognosis in ACLD (*n *= 284)

**Figure 5:**
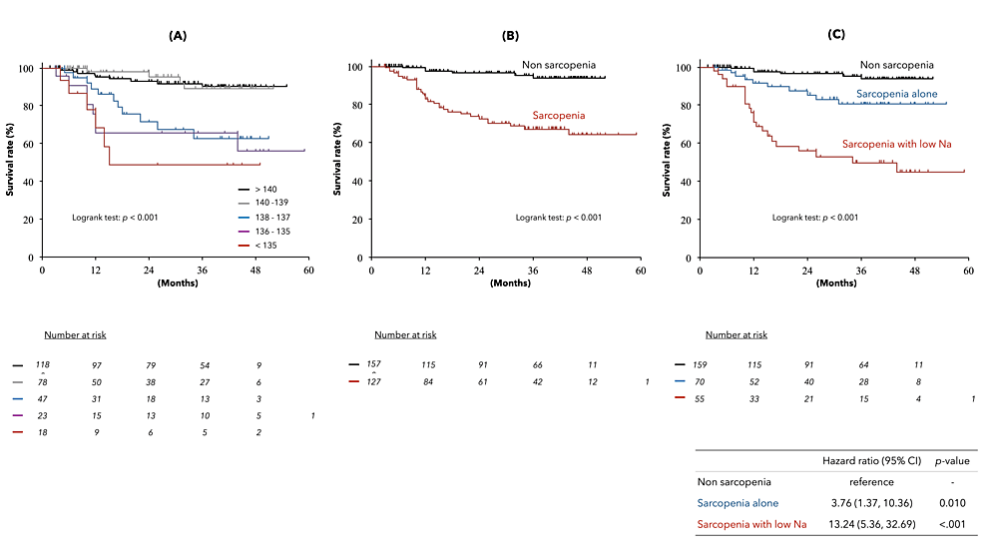
Kaplan–Meier survival curves of the patients with ACLD (median observation period, 32.1 months; 44 liver deaths). The upper panel shows the Kaplan–Meier survival curves for the five groups based on the serum Na level (A) and by the presence of sarcopenia (B). Patients with serum Na levels < 139 mEq/L and patients with sarcopenia had a significantly worse prognosis (log-rank test, *p* < 0.001). Next, the Kaplan-Meier survival curves (C) were generated for three categories of advanced chronic liver disease (ACLD) patients, combining patients with and without sarcopenia and serum Na levels < 139 mEq/L. The prognosis of the patients with sarcopenia was significantly stratified by low serum Na levels.

During a mean interval of 29.6 ± 16.1 (median, 32.1) months, 44 liver-related deaths occurred, including two liver transplants. Of these, 34 were due to liver failure and 10 to HCC. The Kaplan-Meier survival curves in ACLD (Figure 5) showed a significant stratification by serum Na levels (A) and sarcopenia (B). In particular, analysis by the serum Na levels showed significantly lower survival rates in the patients with serum Na level < 139 mEq/L. (C) The Kaplan-Meier survival curves of the three groups of patients with ACLD (no sarcopenia, sarcopenia alone, and sarcopenia combined with serum Na level < 139 mEq/L) were compared. The results showed a significant difference in the prognosis among the three groups.

Factors contributing to prognosis in patients with ACLD

The univariate analysis based on the Cox proportional hazards model was performed for sex, etiology, blood parameters, LS, NLR, MELD-Na score, ALBI score, presence of ascites, sarcopenia, and HCC. A multivariate analysis (Table 4) was performed with five significant factors selected by stepwise increasing or decreasing methods, and the ALBI score and sarcopenia were extracted as independent prognostic factors.

**Table 4:** Prognostic factors by the Cox proportional hazards model in ACLD ALBI, albumin–bilirubin; CRP, C-reactive protein; HCC, hepatocellular carcinoma; LS, liver stiffness; MELD–Na, model for end-stage liver disease sodium; NLR, neutrophil-to-lymphocyte ratio; PT, prothrombin activity; T.Bil, total bilirubin; TC, total cholesterol

	Univariate analysis		Multivariate analysis	
	HR (95% CI)	p-value	HR (95% CI)	p-value
Sex (male)	2.59 (1.25–6.06)	0.010		
Etiology (alcohol)	2.07 (1.03–3.99)	0.034		
T.Bil (mg/dL)	1.34 (1.16–1.50)	0.001		
Albumin (g/dL)	0.20 (0.13–0.30)	< .001		
TC (mg/dL)	0.97 (0.97–0.98)	< .001		
Hemoglobin (g/dL)	0.74 (0.63–0.86)	< .001		
Platelets (×10^4^/mm^3^)	0.85 (0.80–0.91)	< .001	0.95 (0.89–1.01)	0.181
PT (%)	0.95 (0.94–0.97)	< .001		
CRP (mg/dL)	1.27 (1.11–1.41)	< .001		
Sodium (mEq/L)	0.86 (0.81-0.91)	< .001	0.94 (0.87–1.00)	0.109
Sodium (< 139 mEq/L)	4.14 (2.28–7.67)	< .001		
LS (kPa)	1.25 (1.16–1.33)	< .001		
Ascites	11.89 (6.18–23.58)	< .001	1.62 (0.69–3.89)	0.236
Sarcopenia	7.40 (3.32–19.65)	< .001	4.03 (1.70–11.10)	< .001
NLR	1.31 (1.12–1.47)	0.002		
MELD–Na score	6.88 (3.54–13.12)	< .001		
ALBI score	5.37 (3.51–8.23)	< .001	2.60 (1.46–4.66)	< .001
Presence of HCC	4.56 (2.34–8.87)	< .001		

## Discussion

In this study, based on MRE imaging analysis, we found that not only increased LS (i.e., PHT) but also sarcopenia comorbidity were independently associated with decreased serum Na levels in Japanese CLD patients. The impact of these factors was reflected in an unnoticeable, mild decrease in Na levels (<139 mEq/L), which contributed to poor prognosis.

Currently, MRE is considered the most accurate NIT for the diagnosis and staging of liver fibrosis, and <2.5 kPa is considered the normal value of LS on MRE [32]. In previous studies, the cutoff values of LS on MRE for predicting liver-related events have been studied for each etiology. Ajmera et al. [33] noted that the cumulative incidence of liver-related events in patients with NAFLD having LS < 3.63 kPa is very low at 0.5% per year. By contrast, LS thresholds predicting hepatic decompensation range from 5.0 to 6.48 kPa for each etiology [34,35,36,37,38]. In this study, which included multiple etiologies, the prevalence of thrombocytopenia significantly increased at LS ≥ 3.5 kPa, and the prevalence of varices and ascites increased at ≥4.5 and ≥5.1 kPa, respectively (*p* < 0.01). Thrombocytopenia is closely related to the onset and severity of gastroesophageal varices and is considered a simple indicator of PHT in clinical practice [39]. At the Baveno VII Consensus Conference on PHT [40], platelet count < 15 × 10^4^/mm^3^ was listed as one of the exclusion criteria for clinically significant PHT. Ascites is the most frequent first decompression event in cirrhosis, and patients with grade 1 ascites have significantly shorter survival rates than those without ascites [41,42]. That is, LS values reflect the severity of PTH, and our data indicate that LS ≥ 3.5 kPa is the beginning of the risk of PHT and LS ≥ 5.1 kPa is a high risk for hepatic decompensation. These results are consistent with those of previous reports.

To the best of our knowledge, this is the first report analyzing the relationship between serum Na levels and LS measured by MRE in CLD. We classified patients with CLD into five groups based on the serum Na level and investigated the association between lower serum Na levels and LS- and PHT-related events. The results showed that the impact of LS was not observed in patients with serum Na level ≥ 139 mEq/L, and increased LS was significantly associated with decreased Na levels at <139 mEq/L. The cutoff value of LS contributing to serum Na level < 139 mEq/L was ≥3.4 kPa. The incidence of thrombocytopenia, varices, and ascite complications by the serum Na level also showed the same trend, with the incidence of PHT complications significantly increasing with lower serum Na levels only in the range of serum Na levels < 139 mEq/L.The pathogenesis of hyponatremia in cirrhosis is well understood in the decompression phase; however, little is known in the pre-ascite phase. In the present study, we found for the first time that elevated LS strongly affects the onset and progression of PHT and is an independent associated factor with low serum Na levels. It was then clearly demonstrated that the decrease in serum Na level associated with PHT is <139 mEq/L and that the low Na associated with PHT occurs when the level of liver fibrosis is LS ≥ 3.5 kPa.

Complications of sarcopenia are closely associated with poor prognosis in patients with cirrhosis [43]. Gastrointestinal malabsorption and insulin resistance caused by PHT affect malnutrition in cirrhosis, causing sarcopenia. A combination of other factors, such as hormonal abnormalities, micronutrient deficiencies, low branched-chain amino acids, hyperammonemia, and systemic inflammation, are also thought to contribute to sarcopenia in liver diseases [43]. In the present study, a low serum Na level was newly found as a factor associated with sarcopenia in CLD. Interestingly, the association between lower serum Na levels and sarcopenia was observed not only in patients with severe liver fibrosis but also in patients with LS < 3.4 kPa, independent of age and malnutrition. However, even recent reports studying the association between sarcopenia and chronic hyponatremia have so far failed to clarify the presence of confounding factors or which is the primary cause [44,45]. The Na content in the body of the average adult male is estimated to be about 92 g, most of which is found in extracellular fluid (50%) and skeletal muscle (40%), with only 10% in intracellular fluid. In addition, Na is important for skeletal muscle contraction and neuronal excitation [46]. Thus, there may be a direct relationship between the amount of Na in the body and muscle mass. However, further investigation is needed to clarify this hypothesis.

In the present study, both sarcopenia and low Na were associated with poor prognosis in ACLD. A retrospective study of 171 Japanese patients with cirrhosis reported that patients with serum Na levels < 139 mEq/L had a significantly poorer prognosis [47]. Our results are similar and the coexistence of sarcopenia may explain this outcome. The impact of sarcopenia on survival was observed primarily in patients with cirrhosis of Child-Pugh class A-B or low MELD scores [43]. In the present study, serum Na levels significantly stratified the survival of patients with sarcopenia in ACLD, possibly because the degree of decrease in serum Na levels is related to the severity of liver disease and renal dysfunction. Therefore, we consider that serum Na levels should also be added to the assessment of sarcopenia in ACLD. For cirrhosis patients with ascites, almost all guidelines recommend a salt-restricted diet. While a very low-sodium diet does not cause Na deficiency in healthy individuals, strict salt restriction in cirrhosis patients may promote the resolution of ascites, but it may also cause hyponatremia and diuretic-induced renal failure. Furthermore, it has been reported that patients who received diuretics alone without salt restriction had improved hyponatremia and had better outcomes [5,48]. These discrepancies may suggest that some patients may be suitable for salt restriction while others are not. Based on our data showing that low Na levels are associated with the complications and severity of sarcopenia, easy salt restriction in ACLD may actually worsen the prognosis and should be determined on an individual patient basis. We think that salt restriction should be avoided, especially in the elderly and patients with sarcopenia.

This study has several limitations. First, this single-center, backward-looking analysis needs to be validated at other centers. Specifically, only 3% of the patients had hyponatremia because patients with severe ascites or severe disease were not indicated for MRE, since our study included patients who had undergone MRE. Furthermore, regional differences in the rate of hyponatremia in patients with cirrhosis have been noted and need to be validated in other races [47,49]. Second, this study does not confirm that the type of hyponatremia in cirrhosis is dilutional.

## Conclusions

This study showed that lower serum Na levels in CLD, along with PHT, are strongly associated with sarcopenia and affect mortality. These findings suggest that serum Na levels may be a new therapeutic target for sarcopenia and may also be useful in clinical practice to determine the appropriateness of salt restriction for patients with ascites. Our results also indicate the need for further investigation of the relationship between Na-water metabolism and muscle atrophy in CLD.
